# Drag reducing polymers decrease hepatic injury and metastases after liver ischemia-reperfusion

**DOI:** 10.18632/oncotarget.18322

**Published:** 2017-05-31

**Authors:** Samer Tohme, Marina V. Kameneva, Hamza O. Yazdani, Vikas Sud, Julie Goswami, Patricia Loughran, Hai Huang, Richard L. Simmons, Allan Tsung

**Affiliations:** ^1^ Department of Surgery, University of Pittsburgh Medical Center, Pittsburgh, PA, USA; ^2^ McGowan Institute for Regenerative Medicine, University of Pittsburgh, Pittsburgh, PA, USA; ^3^ Department of Bioengineering, University of Pittsburgh, Pittsburgh, PA, USA; ^4^ Center for Biologic Imaging, Department of Cell Biology, University of Pittsburgh, PA, USA

**Keywords:** liver, Ischemia reperfusion injury, drag reducing polymers, liver metastasis, metastatic colorectal cancer

## Abstract

**Introduction:**

Surgery, a crucial therapeutic modality in the treatment of solid tumors, can induce sterile inflammatory processes which can result in metastatic progression. Liver ischemia and reperfusion (I/R) injury, an inevitable consequence of hepatic resection of metastases, has been shown to foster hepatic capture of circulating cancer cells and accelerate metastatic growth. Efforts to reduce these negative consequences have not been thoroughly investigated. Drag reducing polymers (DRPs) are blood-soluble macromolecules that can, in nanomolar concentrations, increase tissue perfusion, decrease vascular resistance and decrease near-wall microvascular concentration of neutrophils and platelets thereby possibly reducing the inflammatory microenvironment. We hypothesize that DRP can potentially be used to ameliorate metastatic capture of tumor cells and tumor growth within the I/R liver.

**Methods:**

Experiments were performed utilizing a segmental ischemia model of mice livers. Five days prior or immediately prior to ischemia, murine colon adenocarcinoma cells (MC38) were injected into the spleen. DRP (polyethylene oxide) or a control of low-molecular-weight polyethylene glycol without drag reducing properties were administered intraperitoneally at the onset of reperfusion.

**Results:**

After three weeks from I/R, we observed that liver I/R resulted in an increased ability to capture and foster growth of circulating tumor cells; in addition, the growth of pre-existing micrometastases was accelerated three weeks later. These effects were significantly curtailed when mice were treated with DRPs at the time of I/R. Mechanistic investigations in vivo indicated that DRPs protected the livers from I/R injury as evidenced by significant decreases in hepatocellular damage, neutrophil recruitment into the liver, formation of neutrophil extracellular traps, deposition of platelets, formation of microthrombi within the liver sinusoids and release of inflammatory cytokines.

**Conclusions:**

DRPs significantly attenuated metastatic tumor development and growth. DRPs warrant further investigation as a potential treatment for liver I/R injury in the clinical setting to improve cancer-specific outcomes.

## INTRODUCTION

Surgery is a crucial intervention which provides a chance of cure for patients with solid tumors. Although surgical excision of primary and metastatic tumors can save or extend life, it has long been acknowledged that the surgical insult itself may precipitate or accelerate tumor recurrence [1]. Sterile inflammation induced by surgery represents the body's response to a perceived non-infectious danger and is fundamental for reparative processes. However, when excessive, this inflammatory response can contribute to severe organ damage and dysfunction and may also enhance the risk of metastatic progression [1, 2].

When feasible, resection of hepatic colorectal metastases promotes improved survival compared with chemotherapy alone [3]. Unfortunately, hepatic recurrence after surgical resection occurs in 50-60% of patients and is the major cause of treatment failure [4]. There is growing experimental and clinical evidence that surgery aimed to resect metastatic colorectal cancer to the liver can induce formation of new metastatic foci and growth of micrometastatic disease [1, 5]. During surgery, the liver is routinely subjected to ischemic injury due to mechanical manipulation or interruption of the hepatic blood supply that is necessary to control blood loss. Moreover, further damage occurs from excessive activation of inflammatory pathways following restoration of blood flow [6, 7]. Such damaging effects contribute to liver ischemia reperfusion (I/R) injury, which can have a significant impact on postoperative outcomes [8, 9]. Additionally, there are growing concerns surrounding the role of hepatic I/R injury in the oncological setting. Previous studies have clearly demonstrated that the inflammation caused by liver I/R in animal models can stimulate tumor cell adhesion, promote the incidence of metastasis formation, and accelerate the outgrowth of pre-existing hepatic micrometastases [5, 10, 11]. Indeed, in patients undergoing resection for liver tumors, longer ischemic times are associated with accelerated recurrences following attempted curative resection [12].

Experimental studies have elucidated some of the dominant molecular pathways important in the pathogenesis of liver I/R and their role in fostering both the capture of circulating tumor cells and their metastatic growth [13]. Neutrophils and platelets are the front-line defense cells against injury and have long been implicated as among the principal cells in the innate immune response induced by hepatic I/R [13–18]. We have recently shown that neutrophils can contribute to metastatic tumor growth after liver I/R by either enhancing establishment of metastatic foci or promoting the growth of existing micrometastatic disease [5]. Similarly, platelets may play an important role in the generation of sinusoidal microthrombi capable of arresting circulating cancer cells leading to new metastatic foci [19, 20]. Ameliorating injury by targeting these pathways in experimental models, however, has not yet been translated into clinically effective approaches [13]. Therefore, it is of potential value to seek ways of avoiding liver I/R mediated injury and thereby improve oncologic outcomes.

Drag Reducing Polymers (DRPs) are long-chain, viscoelastic, blood soluble macromolecules with molecular weight over one million Da and a relatively linear structure. In the mid-20^th^ century, Toms described the ability of small concentrations of soluble macromolecules DRPs to reduce resistance to turbulent flow in a pipe [21]. DRPs have since been extensively used in industry [22]. Recently, the potential benefits of apparently non-toxic and degradable DRPs have been tested in a number of experimental models of ischemia. Even nanomolar concentrations of DRPs in the circulation can improve tissue perfusion and oxygenation and decrease vascular resistance with minimal changes in blood pressure in animal models [22]. DRPs have been shown to improve myocardial perfusion in models of coronary stenosis, reduce the progression of atherosclerosis, protect against pulmonary hypertension, decrease mortality after hemorrhagic shock and reduce foreign body reaction to implants [23–27]. Circulating DRPs increase the concentrations of RBCs along the vessel wall and decrease the margination of leukocytes and platelets in the microcirculation [22, 28]. This rheological effect thereby reduces the interactions between platelets, leukocytes and the vascular endothelium essential for maximal inflammatory infiltration [25]. Since both hypoxia and inflammation enhance tumor growth, the present experiments were designed to investigate the role of DRP on both metastatic capture of circulating tumor cells and on the growth of recently established hepatic micrometastases.

## MATERIALS AND METHODS

### Animals

Male wild-type (C57BL/6) mice (8-10 weeks-old) were purchased from Jackson Laboratories. Animal protocols were approved by the Animal Care and Use Committee of the University of Pittsburgh.

### DRP treatment

DRP (Polyethylene oxide (PEO), 4 million Da MW, Sigma-Aldrich®) was given to animals intraperitoneally at various concentrations. Each polymer preparation was tested for drag reducing and rheological properties as previously described [24]. Control animals were treated with the same chemical but with significantly lower MW (PEG, polyethylene glycol, 1000 Da MW, Sigma-Aldrich®), which does not have drag-reducing properties.

### Liver I/R model

A nonlethal model of segmental (70%) hepatic warm ischemia and reperfusion was used [22, 29]. DRP (100 μl, concentrations 10 to 1000 ppm per mouse) or PEG (100 μl, 1000 ppm) were injected intraperitoneally immediately after ischemia. Sham animals underwent anesthesia, laparotomy, and exposure of the portal triad without hepatic ischemia.

### Metastases models

The first set of metastases experiments were designed to evaluate whether I/R increased tumor growth in a model of circulating micrometastases. Colorectal liver metastases were induced in mice as previously described [5]. In brief, at the time of reperfusion 5×10^4^ MC38 cells (colorectal cancer cell lines) were injected into the spleen using a 27-gauge needle. At the same time, mice received either DRP or PEG intraperitoneally. Tumor cells were allowed to circulate for 10 minutes before splenectomy. The next set of experiments was designed to evaluate whether DRP protects from I/R-induced increased growth of already established micrometastatic disease. In these experiments, tumor cells were injected into the spleen through a small left lateral flank incision followed by splenectomy. Micrometastases were allowed to develop throughout the liver for 5 days. Then the mice underwent either hepatic I/R with DRP or PEG injection as described above.

### Statistical analysis

Results are expressed as the mean ± standard error of mean (SEM). Group comparisons were performed using ANOVA and Student's *t*-test. A *p* < 0.05 was considered statistically significant.

## RESULTS

### DRPs protect from liver I/R injury

**Figure 1:**
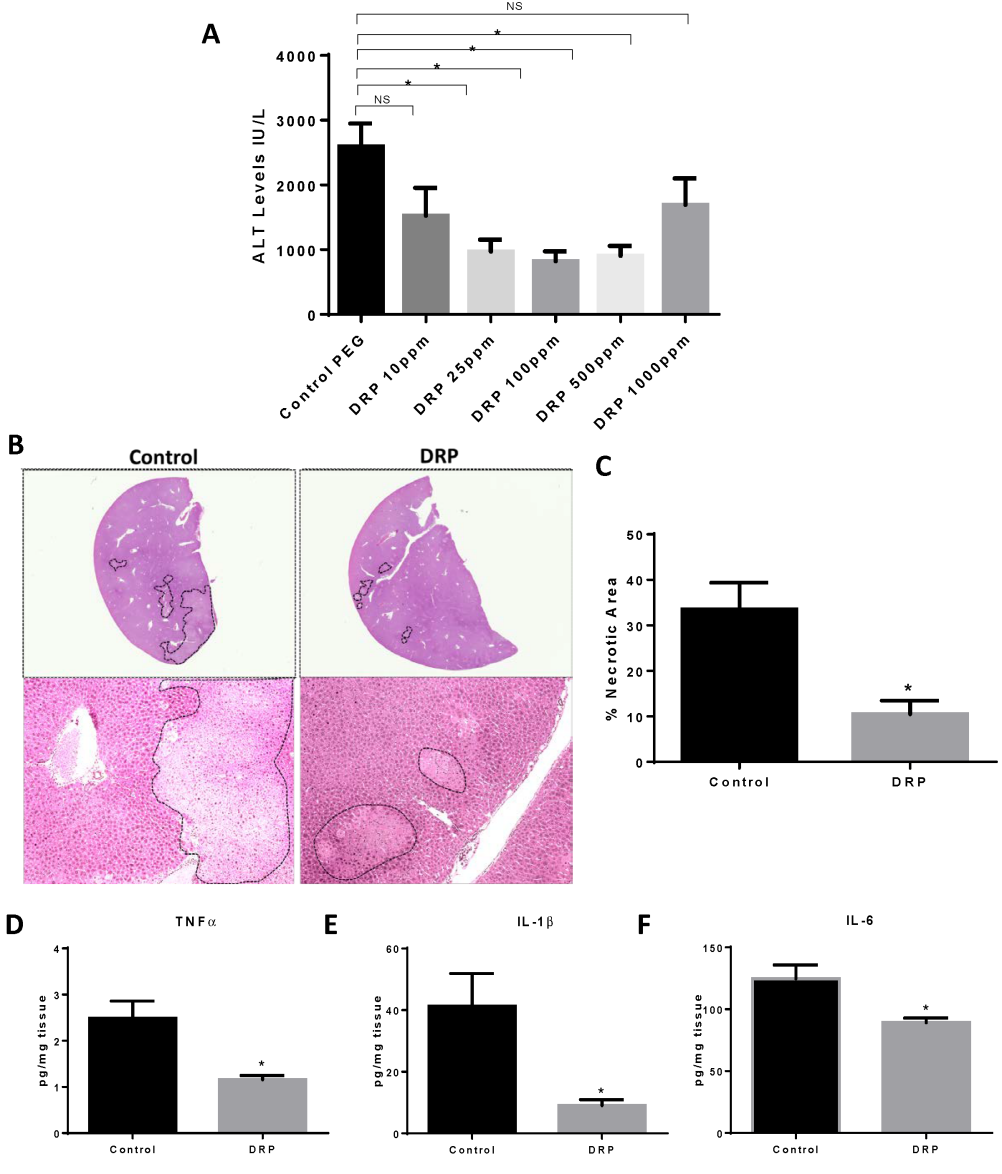
DRPs protect from liver I/R injury **A**. I/R-treated mice were given different concentrations of DRPs or control PEG intraperitoneally at the time of reperfusion. Serum ALT levels were assessed after 1 hour of ischemia and 6 hours of reperfusion. Data represent the mean ± SE (*n* = 6 mice/group). **B**. Representative H&E stained liver sections showing decreased areas of hepatic necrosis in I/R-treated mice receiving DRP. **C**. Quantification of necrotic hepatocytes in H&E stained liver sections from control or DRP-treated mice 6 hours after reperfusion. **D**., **E**. and **F**. DRPs decrease I/R-induced inflammatory cytokine levels. Liver tissue levels of TNF-α, IL-1β and IL-6 obtained from control and DRP-treated mice at 6 h after reperfusion were measured by ELISA. *P < 0.05, NS: not significant.

We first sought to evaluate the role of different concentrations of administered DRPs on global liver injury after 6 hours of reperfusion. DRPs significantly reduced liver damage as evidenced by reduced serum ALT levels compared with mice receiving PEG in doses as low as 25 ppm and as high as 500ppm; ALT levels were not significantly reduced at 1000 ppm (Figure 1A). A DRP concentration of 100ppm was used for the rest of the experiments as it provoked the least amount of ALT release after I/R without visible effect on mice. The intraperitoneal route was chosen to allow more gradual and prolonged direct access to the venous circulation via the diaphragmatic lymphatics without prolonging anesthesia needed for slow intravenous infusion of the viscoelastic DRPs. Histology was consistent with the serum ALT levels of liver damage, with the presence of severe sinusoidal dilatation and confluent pericentral hepatocellular necrosis in liver tissue from control mice but not from DRP treated-mice (Figure 1B and Figure 1C). In addition, the liver tissue levels of the proinflammatory cytokines, TNFα, IL-1β and IL-6 were significantly lower in recipients of DRPs compared with control mice subjected to liver I/R (Figure 1D and Figure 1E and Figure 1F).

### DRPs decrease neutrophil infiltration and neutrophil extracellular trap (NET) formation after I/R

**Figure 2:**
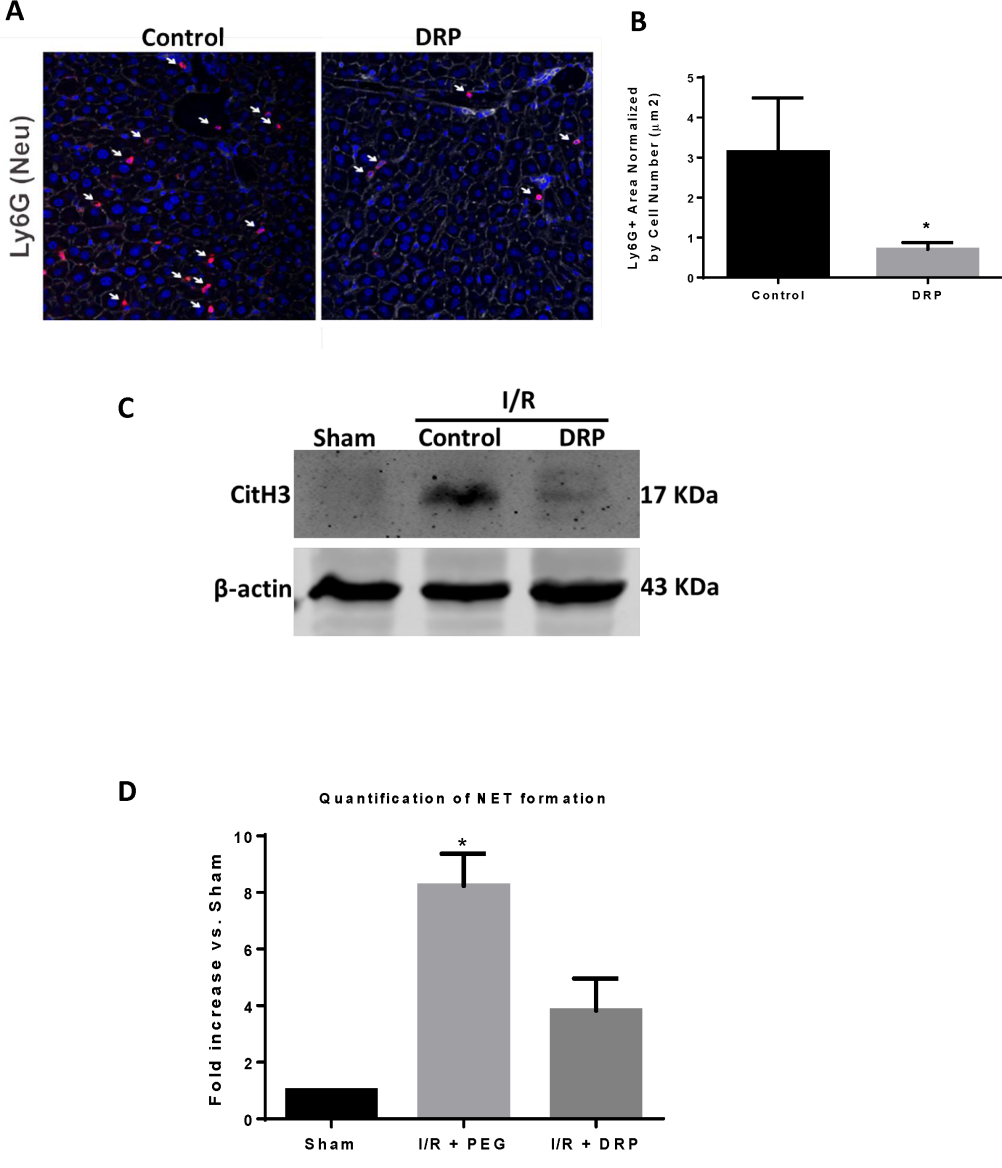
DRPs decrease neutrophil infiltration and neutrophil extracellular trap formation after I/R **A**. and **B**. Using confocal microscopy, there is a significant decrease in infiltrating neutrophils 6 hours after mice were subjected to I/R in DRP treated mice compared to mice that received control (mean 0.8 μm^2^ Ly6G^+^ area/total cells versus 3.1 μm^2^ Ly6G^+^ area/total cells, *p* < 0.001). Ly6G (red), nuclei (blue). Scale Bars 100μm. **C**. Cit-H3 protein levels were determined by Western blot in sham, I/R + control PEG, and I/R + DRP mice groups 6 hours after liver I/R. **D**. NETs acutely form in liver tissue 6 hours after liver I/R as assessed by serum levels of MPO-DNA. Treatment with DRPs after liver I/R resulted in a significant decrease in the levels of serum MPO-DNA at 6 hours. Results are expressed as the relative folds increase of MPO-DNA levels compared with sham; mean±SEM (*n* = 6/group). *P < 0.05.

We have previously shown that liver I/R induced increases in ALT and liver necrosis accompanied by increases in hepatic neutrophil infiltration and intrahepatic NET formation [30]. We therefore examined whether the protective effects of DRPs was related to a decrease in neutrophil influx and NET formation in the livers. Figure 2A and 2B show that there was a significant decrease in infiltrating neutrophils within the ischemic liver lobes in the mice treated with DRPs compared to liver lobes from mice treated with PEG. Ischemic lobes also exhibited significantly higher levels of citrullinated-histone H3, a specific marker of NET formation, after liver I/R which was significantly reduced when mice were treated with DRPs (Figure 2C). Furthermore, we found that the serum level of MPO-DNA complexes, a marker that circulating nucleosomes are derived from NET formation, was significantly decreased in mice undergoing liver I/R with DRP treatment compared to controls (Figure 2D).

### DRPs decrease platelet aggregation and micro thrombi formation in livers after I/R

**Figure 3:**
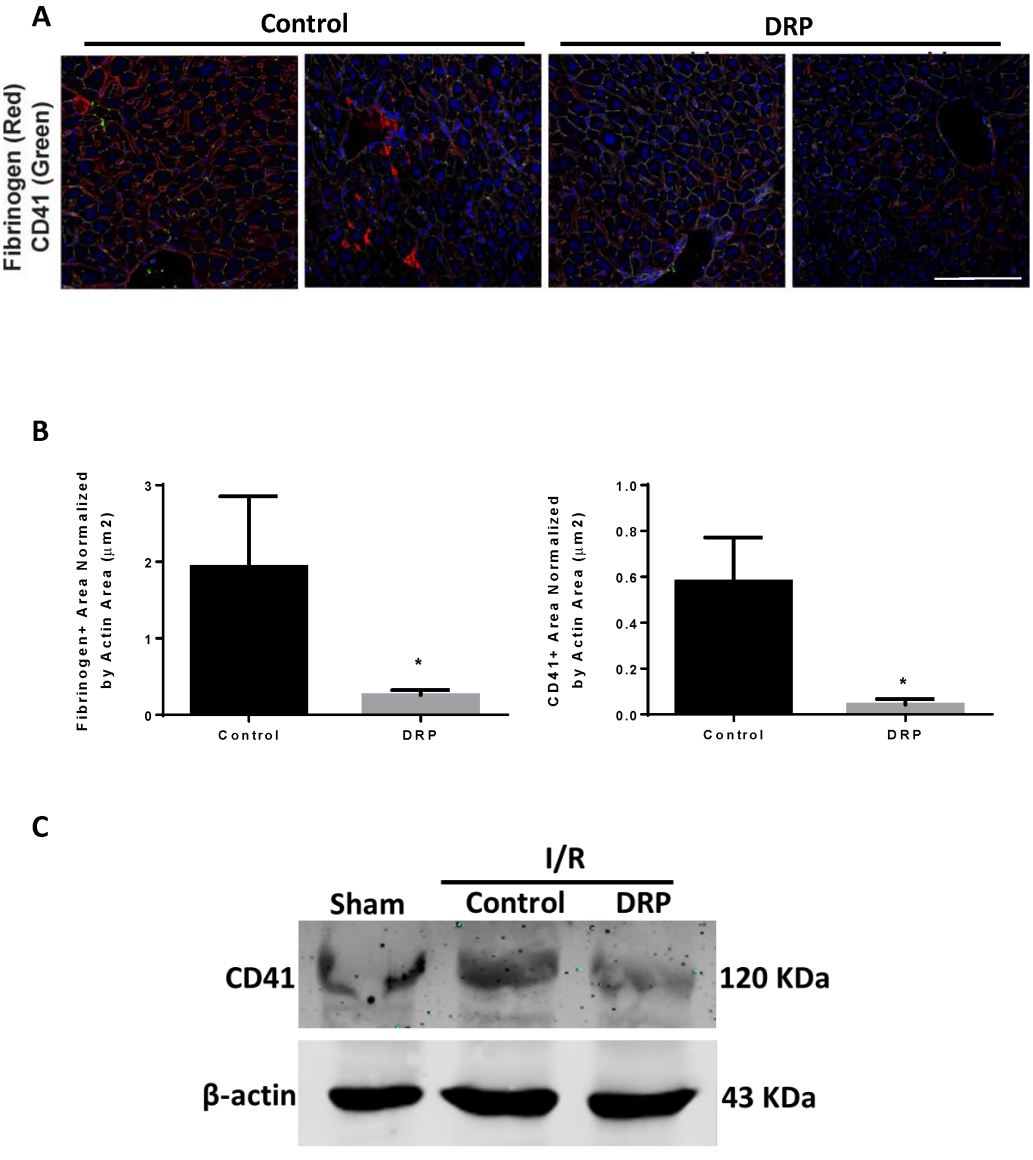
DRPs decrease platelet aggregation and micro thrombi formation in liver after I/R **A**. and **B**. Representative immunofluorescence images by confocal microscopy of mice liver sections showing decreased platelet aggregation and microthrombi formation in mice treated with DRP after liver I/R versus mice treated with control (mean 0.83 μm^2^ versus 1.97 μm^2^ Fibrinogen^+^ area/Actin area (μm^2^), *p* < 0.001 and mean 0.03 μm^2^ versus 1.57 μm^2^ CD41^+^ area/Actin area (μm^2^), *p* < 0.001. Fibrinogen (red), CD41 (green), nuclei (blue). Scale Bars 100μm. **C**. CD41 level was increased in the hypoxic liver tissue in mice subjected to I/R compared to sham. CD41 levels were decreased in DRP-treated mice after liver I/R.

Examination of liver sections of hepatic lobes 6 hours after I/R demonstrated a significant increase in platelet aggregation within liver sinusoids and a significant increase in fibrin-rich microthrombi formation in mice treated with control PEG compared to DRPs (Figure 3A and 3B). In similar findings, DRPs significantly decreased platelet sequestration within the hepatic lobes exposed to I/R as seen on Western Blots examining the expression of CD41, a platelet marker (Figure 3C).

### DRPs decrease the formation of new liver metastases after liver I/R

**Figure 4:**
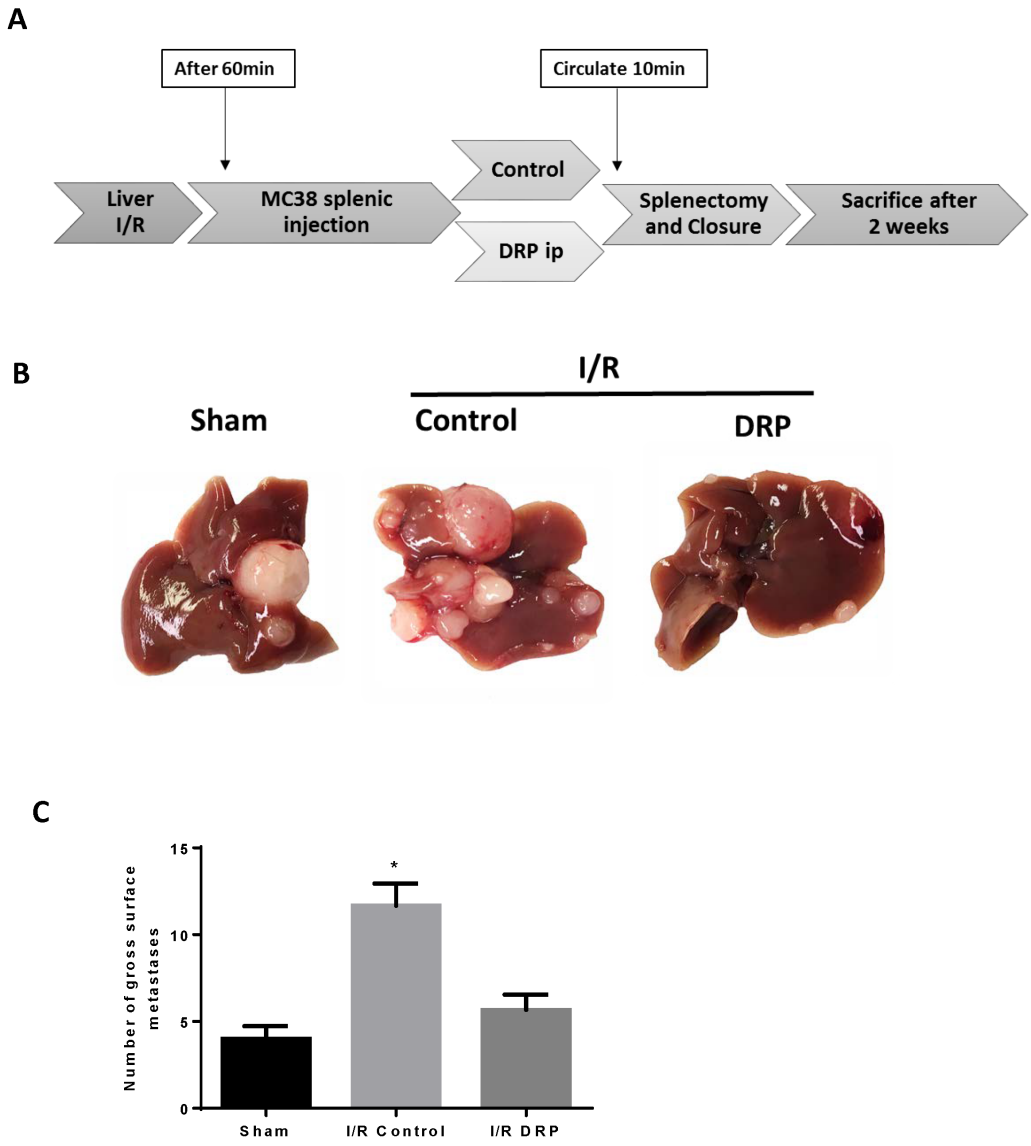
DRPs decrease the formation of new liver metastases after liver I/R **A**. A schematic representation of the experimental design is depicted. Mice were subjected to liver I/R in order to induce surgical stress. Intrasplenic injection of MC38 colorectal cancer cell lines was performed at the same time. DRPs or control PEG intraperitoneally were given at the time of procedure. **B. and C**. After three weeks, I/R resulted in a significant increase in gross surface metastatic nodules compared with the sham groups (mean 12 nodules in I/R versus 4 nodules in sham; *p* < 0.001). Treatment with DRPs resulted in a significant decrease in the number of gross metastases compared to control treated mice (mean 12 nodules in I/R control versus 5 nodules in I/R DRP; *p* < 0.001).

We next aimed to mimic the surgical setting in which major resection of livers in cancer patients with circulating tumor cells foster metastatic spread to the remaining liver [31]. We have previously shown that liver I/R in mice creates a niche favorable for the capture and growth of metastases of circulating colon cancer cells [5]. The presence of NETs within the liver lobes exposed to I/R have been shown to provide conditions under which circulating cancer cells are captured and promote formation of metastases. In addition, the presence of microthrombi have the potential to increase the probability of the tumor cell to arrest in the hepatic microcirculation and establish metastatic foci. Having shown that DRPs decrease NET and microthrombi formation after liver I/R, we sought to determine whether DRP can decrease the development of new metastases. The experimental protocol is illustrated in Figure 4A. MC38 cells were injected into the spleen immediately after a 60-minute period of lobar I/R. Recipients of tumor cells and cohort controls were given DRPs or PEG, respectively, at the time of reperfusion and tumor injection. Quantification of gross hepatic metastases was performed when the mice were sacrificed at 3 weeks. Figure 4B shows that livers of mice subjected to I/R without DRPs treatment contained significantly more hepatic metastases compared with mice not receiving I/R (mean 13 nodules in I/R versus 2 nodules in sham; *p* < 0.001). Administration of DRPs after liver I/R resulted in a 69% reduction in tumor nodules compared with untreated I/R mice at 3 weeks (mean 4 nodules, *p* = 0.003) (Figure 4C).

### DRPs halts the I/R-induced accelerated growth of established liver micrometastases

**Figure 5:**
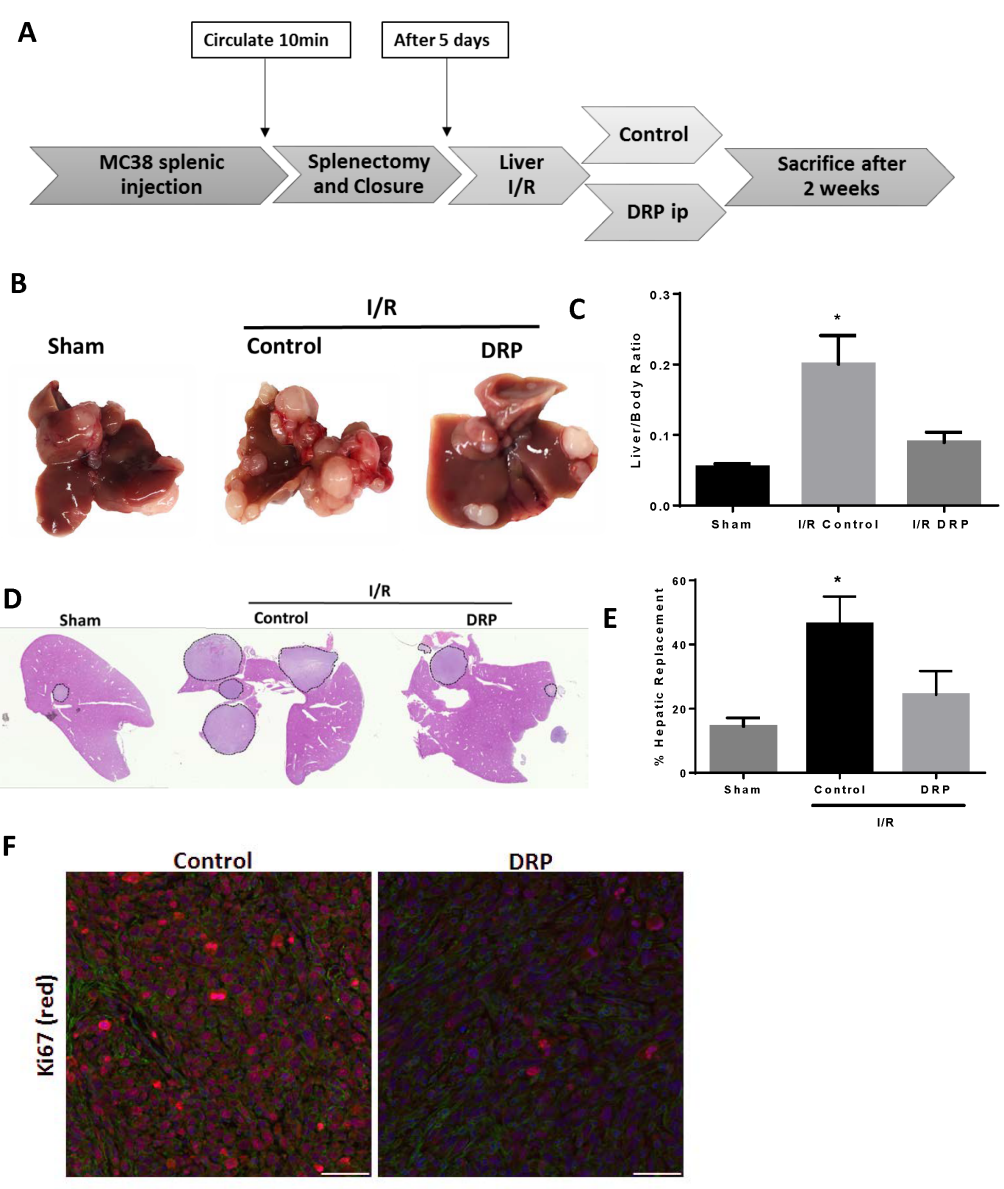
DRPs halt the I/R-induced accelerated growth of established liver micrometastases **A**. Schematic representation of the experimental design is depicted. Intrasplenic injection of MC38 cells was performed and metastatic tumor was allowed to grow for 5 days before the mice were subjected to liver I/R. DRPS or control PEG were given at the time of liver I/R. At 3 weeks, the mice were sacrificed and tumor growth was assessed. **B**. Representative images of hepatic nodules after necropsy in mice subjected to sham or I/R with or without DRP treatment. **C**. Liver I/R resulted in a significant increase in tumor growth at three weeks compared to sham mice seen by liver-to-body ratio. Treatment with DRPs after I/R resulted in a significant decrease in growth of already established micrometastases. **D**. and **E**. DRP treatment resulted in a significant decrease in tumor burden at three weeks as seen by percentage hepatic replacement by metastatic tumor. **F**. Treatment with DRPs during I/R significantly attenuated tumor cell proliferation three weeks later as seen by decreased Ki67 staining (mean 2.62 Ki67^+^ nuclei/ Area Actin 10^5^μm^2^ I/R control versus 8.12 Ki67^+^ nuclei/10^5^μm^2^ in I/R DRP group, *p* < 0.001). Ki67 (red), nuclei (blue), actin (green). Scale Bars 100μm.

**Figure 6:**
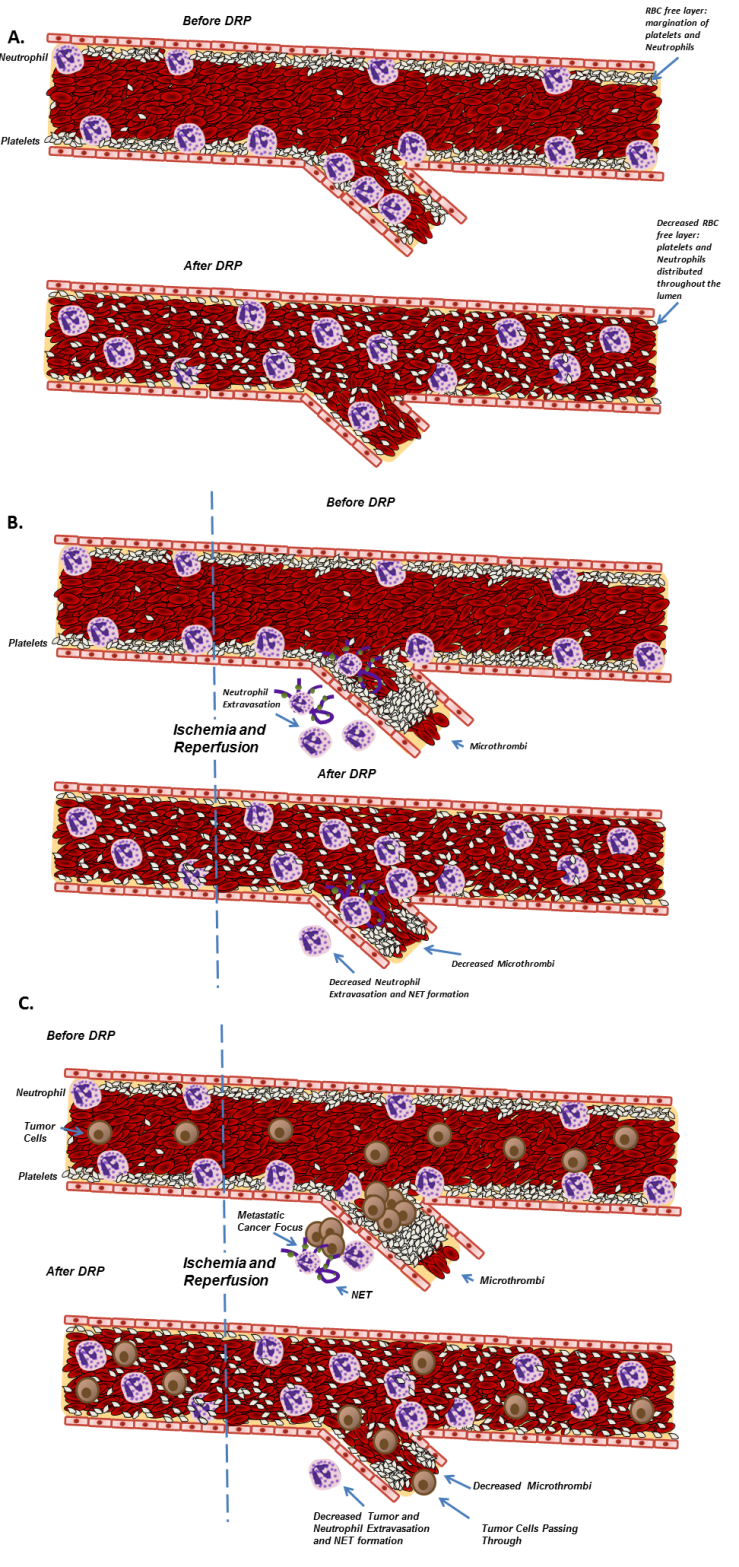
Schematic diagram illustrating the proposed role of DRPs in decreasing injury and metastases after liver I/R **A**. DRPs alter the traffic of blood cells within microvessels. **B**. DRPs attenuate the acute inflammatory response, decrease the recruitment and activation of neutrophils and concurrent NET formation, decrease the formation of microthrombi and subsequently protect the liver from the resulting damage. **C**. DRPs have favorable oncological outcomes in mice subjected to surgical stress by decreasing both the formation of new metastatic disease and the growth of established micrometastases.

We have previously shown that established micrometastatic tumors manifest accelerated growth in liver lobes exposed to I/R, an effect partially reversed by inhibition of inflammation associated with NET formation [5]. We used our model to examine whether DRPs, shown above to reduce the protumorigenic proinflammatory cytokine response and NET formation, can affect the growth of these existing micrometastases. MC38 cells were injected into the spleen and micrometastases were allowed to develop. Five days later, the mice were subjected to I/R surgery and treated with PEG (as a control) or with DRPs (Figure 5A). Mice treated with DRP displayed significantly decreased tumor growth, which was grossly appreciable as smaller and less numerous tumors (Figure 5B). In addition, DRP significantly decreased the tumor load after I/R as evidenced by the liver-to-body weight ratio and the tumor hepatic replacement area (Figure 5C, 5D and 5E). Tumors from mice treated with DRP after I/R showed a significant decrease in proliferation compared to PEG-treated mice subjected to I/R as evident by decreased Ki6 staining (Figure 6F). The results obtained from these two models (Figure 5A and Figure 6A) demonstrate that hepatic I/R has a stimulatory effect on tumor cell capture and growth of metastases and that is significantly reduced by using DRPs.

## DISCUSSION

During the surgical resection of metastatic cancer in the liver, some degree of liver I/R injury is inevitable and is associated with worse short term and long term outcomes [13]. We originally hypothesized that the administration of minute quantities biocompatible DRPs to the circulation might alter the tendency of the liver to capture circulating potentially metastatic colon cancer cells. This hypothesis was based on the well documented rheological properties of DRPs during blood flow which has been shown to improve tissue perfusion and oxygen delivery in experimental models. In addition, a novel biorheological effect of DRPs is their ability to alter the distribution of innate inflammatory cells which normally crawl or roll along the endothelial surface within the microvasculature. Under normal physiological conditions, there is a differential distribution of blood cells where RBCs preferentially flow in the core of blood vessels whereas leukocytes and platelets tend to flow at the marginal plasma layer. This platelet and leukocyte rich layer adjacent to the endothelium has traditionally been labelled as the near-wall “cell-free”, i.e. RBC free, layer [32]. When DRPs are added to flowing blood, the RBCs become relatively equally concentrated across the vessel and eliminate margination of the leukocytes and platelets near the endothelium distributing all blood cells evenly. By potentially reducing the hypoxic insult of liver I/R and lowering the marginating tendency of neutrophils and platelets and interfering with the ligand/receptor interactions between these innate inflammatory cells necessary for their extravasation into the I/R injured tissue, we hypothesized that the downstream pro-tumorigenic, both hypoxic and pro-inflammatory, consequences would be then mitigated (Figure 7 outlines a proposed mechanism based on the known properties of DRP).

Indeed, a single intraperitoneal injection of DRP reduced both the number of and bulk of metastatic colon cancer foci resulting from tumor cells injected at the time of liver I/R, but also tumors arising from tumor cells injected 5 days prior to I/R. Coincident with the inhibition of tumor growth in these models, the inflammatory changes induced by I/R was profoundly depressed. There was less liver cell damage, less necrosis, suppressed levels of inflammatory cytokines, less neutrophil infiltration, sinusoidal thrombosis and NET formation. Taken together, the protumorigenic microenvironment was suppressed.

Although the *in vivo* effects of DRPs are incompletely understood, they act primarily in the microvasculature to reduce microturbulence at bifurcations and significantly increase near-wall blood flow velocity [33]. As a consequence, DRPs significantly increase blood flow, increase the number of functioning capillaries and increase microvascular flow volume [34–36]. As a consequence, oxygen delivery and consumption is increased, ischemic injury is reduced in models of myocardial and peripheral vascular insufficiency and survival after hemorrhagic shock is increased. Taken together the reduction of protumorogenic and proinflammatory effects of hypoxia during I/R may contribute one component to the slowing of tumor growth in our model.

Furthermore, when circulating tumor cells are present, DRPs would be expected to reduce the interactions between tumor cells, platelets and neutrophils by mixing them with the main RBC flow. Since bonds between tumor cells, platelets and neutrophils are known to actively facilitate the metastatic process [20, 37], DRPs in the microcirculation, by disturbing this interaction, might hypothetically be expected to minimize the capture of circulating tumor cells. But DRPs also succeed in reducing the growth rate of pre-existing micrometastases suggesting that DRPs alter the microenvironment in which tumors prosper. We have previously shown in our model that both intrahepatic tumor capture and growth are fostered by tissue inflammation and especially NET formation. NETs in turn interact with platelets to promote sinusoidal thrombosis [38–40]. DRPs are shown in our experiments to reduce both NET formation and sinusoidal thrombosis thereby impeding the vicious cycle of hypoxia, inflammation and tumor promotion [30, 39–41].

The role of DRPs in reducing neutrophil diapedesis into I/R tissue reinforces the known importance of NET formation as a critical step in the regulation of tumor growth. NETs are important in various infectious and sterile inflammatory processes [5, 42, 43]. For instance, NETs have been implicated in exacerbating injury in myocardial ischemia and shown to be an important trigger in the formation of atherosclerotic plaques [44, 45]. Both processes are inhibited by small concentrations of DRP which alter shear stress to improve oxygenation and reduce inflammation and possibly also decrease NET formation in the studied systems [26, 27, 34, 35].

These studies were prompted by the clinical problem of increased tumor growth in livers manipulated and thus rendered temporarily ischemic during surgery for cancer. For patients with metastatic or primary liver cancer, liver resection is the primary, though imperfect, means of control. Surgery inevitably creates areas of liver ischemia and reperfusion. It has long been recognized that surgical removal of malignancies may enhance the risk of tumor recurrence [1]. There are few clinically applicable interventions to counteract this phenomenon since the exact mechanisms behind it remain incompletely understood. In the recent years more insight into this phenomenon have been uncovered implicating the role of the proinflammatory cytokines in accelerating the growth and turning on the angiogenic switch in dormant micrometastases [1]. Also, NETs have been shown to promote tumor capture and formation of new metastatic foci [5]. Thus, as hypothesized, DRPs given at the time of tumor injection significantly decreased the development and growth of metastases. DRPs are effective at extremely low concentration and undergo rapid degradation *in vivo* so that the rheological effects are lost within a few days without evident toxicity. Therefore, this is the first study to implicate a role for DRPs as significant therapeutic agent to target the perioperative window to halt the progression of metastases initiated by surgical trauma.

In our experiments, DRPs exhibit an anti-metastatic effect by the combined effects of several possible mechanisms: by improving oxygenation, attenuating pro-tumorigenic pro-angiogenic cytokine interactions, diminish NET formation and decrease microthrombi.

In conclusion, this study demonstrates the novel finding that acute intraperitoneal administration of minute concentrations of DRP to mice significantly protected the liver from I/R injury. In addition, DRPs significantly inhibited I/R-induced promotion of metastases development and growth. The clinical implications of our findings are significant as DRPs may offer a powerful novel approach to the treatment of liver I/R injury and perioperative administration of DRPs could improve both short postoperative and long oncologic outcomes.

## SUPPLEMENTARY MATERIALS AND METHODS


