# Supplementary material for: Drag reducing polymers decrease hepatic injury and metastases after liver ischemia-reperfusion
Source: Oncotarget. 2017 May 31;8(35):59854–66. doi: 10.18632/oncotarget.18322 (PMC5601784; doi:10.18632/oncotarget.18322)
Supplement: Supplementary file 1 [file oncotarget-08-59854-s001.pdf]

Drag reducing polymers decrease hepatic injury and metastases after liver ischemia-reperfusion

Supplementary Material

Assessment of Liver Damage

Liver damage was assessed 6 hours after I/R. Serum alanine aminotransferase (ALT) levels were measured using the DRI-CHEM 4000 Chemistry Analyzer System (HESKA). The extent of parenchymal necrosis in the ischemic lobes was evaluated using H&E stained histological sections, as previously described [1].

Tumor Burden Analysis

Mice were sacrificed three weeks after liver I/R and the livers were harvested. Liver and mice weights were recorded and gross metastatic surface nodules were counted. Intrahepatic tumor burden was assessed by calculating the percentage of hepatic tissue replaced by tumor i.e. the hepatic replacement area (HRA). On H&E stained histological sections at 40x magnification, the area occupied by tumor was quantitatively assessed by Image J (NIH). Results were presented as the mean of the percentage of tumor occupying area (mm^2) with respect to the total area examined (mm^2).

Western Blot

The reagents and antibodies used for Western Blots were as follows: anti-citrullinated histone H3 (Abcam), anti-cd41 (BD Biosciences); β-actin (Sigma). Protein extraction from liver tissue and Western blot analysis were performed following a standard protocol as described previously [2].

Confocal microscopy

For immunofluorescence staining, liver sections were fixed, stained, and imaged using confocal microscopy as previously described [3]. The specific primary antibodies used are as follows: Ly6G (1:100, BD Bioscience), CD41 (1:200, Abcam), fibrinogen (1:500, Abcam) or Ki67 (1:100, Abcam). All slides were scanned under the same conditions for magnification, exposure time, lamp intensity and camera gain. Confocal images were acquired with a PlanApo N (×20 with and without a 2.0 digital zoom)

Quantification of NETs using MPO-DNA

To quantify NETs in mouse sera collected 6 hours after reperfusion, a capture ELISA myeloperoxidase (MPO) associated with DNA was performed as described previously [3].

References

[1] Y. Gu, O. Dirsch, U. Dahmen, Y. Ji, Q. He, H. Chi*, et al.*, "Impact of donor gender on male rat recipients of small-for-size liver grafts," *Liver Transpl.,* vol. 11, pp. 669-678, 6/2005 2005.

[2] A. Tsung, R. Sahai, H. Tanaka, A. Nakao, M. P. Fink, M. T. Lotze*, et al.*, "The nuclear factor HMGB1 mediates hepatic injury after murine liver ischemia-reperfusion," *J.Exp.Med.,* vol. 201, pp. 1135-1143, 4/4/2005 2005.

[3] H. Huang, S. Tohme, A. B. Al-Khafaji, S. Tai, P. Loughran, L. Chen*, et al.*, "Damage-associated molecular pattern-activated neutrophil extracellular trap exacerbates sterile inflammatory liver injury," *Hepatology,* vol. 62, pp. 600-14, Aug 2015.
